# Sorafenib induces cathepsin B-mediated apoptosis of bladder cancer cells by regulating the Akt/PTEN pathway. The Akt inhibitor, perifosine, enhances the sorafenib-induced cytotoxicity against bladder cancer cells

**DOI:** 10.18632/oncoscience.147

**Published:** 2015-03-23

**Authors:** Consuelo Amantini, Maria Beatrice Morelli, Matteo Santoni, Alessandra Soriani, Claudio Cardinali, Valerio Farfariello, Anna Maria Eleuteri, Laura Bonfili, Matteo Mozzicafreddo, Massimo Nabissi, Stefano Cascinu, Giorgio Santoni

**Affiliations:** ^1^ School of Biosciences and Veterinary Medicine, University of Camerino, Camerino, Italy; ^2^ School of Pharmacy, Experimental Medicine Section, University of Camerino, Camerino, Italy; ^3^ Department of Molecular Medicine, Sapienza University, Rome, Italy; ^4^ Department of Medical Oncology, Polytechnic University of Marche, Ancona, Italy

**Keywords:** sorafenib, bladder cancer cells, cathepsin B, Akt, TKI, perifosine

## Abstract

Sorafenib, a tyrosine kinase inhibitor, has been demonstrated to exert anti-tumor effects. However, the molecular mechanisms underlying its effects on bladder cancer remain unknown.

Here, we evaluated the mechanisms responsible for the sorafenib-induced anti-tumor effects on 5637 and T24 bladder cancer cells. We demonstrated that sorafenib reduces cell viability, stimulates lysosome permeabilization and induces apoptosis of bladder cancer cells. These effects are dependent by the activation of cathepsin B released from lysosomes. The sorafenib-increased cathepsin B activity induced the proteolysis of Bid into tBid that stimulates the intrinsic pathway of apoptosis characterized by mitochondrial membrane depolarization, oxygen radical generation and cytochrome c release. Moreover, we found that cathepsin B enzymatic activity, induced by sorafenib, is dependent on its dephosphorylation via PTEN activation and Akt inactivation. Pretreatment with orthovanadate rescued bladder cancer cells from apoptosis. In addition, the Akt inhibitor perifosine increased the sensitivity of bladder cancer cells to sorafenib-induced cytotoxicity.

Overall, our results show that apoptotic cell death induced by sorafenib in bladder cancer cells is dependent on cathepsin B activity and involved PTEN and Akt signaling pathways. The Akt inhibitor perifosine increased the cytotoxic effects of sorafenib in bladder cancer cells.

## INTRODUCTION

Sorafenib is an oral multi-target inhibitor of the Raf-1 and B-Raf kinases, which are members of the Raf/MEK/ERK signaling pathway. In bladder cancer (BC) cells, sorafenib has been found to induce apoptosis, however the cellular and molecular mechanisms responsible for the anti-tumor effects in BC cells remain at present unknown [1]. Sorafenib has been found to inhibit several receptor tyrosine kinases (RTKs) that are involved in neo-angiogenesis and tumor progression, such as vascular endothelial growth factor receptors (VEGFR1-3), platelet-derived growth factor receptors (PDGFR a and b), tyrosine protein kinase Kit (c-kit), fms-related tyrosine kinase 3 (Flt3), Colony stimulating factor 1 receptor (CSF-1R) and Rearranged during Transfection (RET) [2]. *In vivo*, sorafenib has been administered in phase II clinical trials to patients with urothelial carcinoma (UC) who had been treated with one prior chemotherapy regimen [3] and to patients with metastatic UC [4]. In addition, sorafenib has been administered in combination with cisplatin and gemcitabine to patients with node-negative transitional BC cells [5], but no objective responses have been detected to date. Chemotherapy of bladder cancers represents the mainstay of treatment and confers survival advantage. However, despite such advances, it remains disappointing because of its toxicity, reinforcing again the rationale for the discovery of new targeted therapeutic approaches [6].

The phosphoinositide 3-kinase/protein kinase B (PI3-K/Akt) pathway, frequently altered in BC, plays a key role in the regulation of cell proliferation, angiogenesis and cell survival and represents a potential therapeutic target [7,8]. In addition to targeting RTKs, sorafenib also affects the PI3-K/Akt pathways and their negative regulator, the lipid phosphatase and tensin homolog (PTEN) pathway. The activation of PI3K/Akt signaling mediates the acquired resistance to sorafenib in hepatocellular carcinoma (HCC) cells [9]; PTEN over-expression enhances the sensitivity of HCC cells to the anti-proliferative and pro-apoptotic effects of sorafenib [10]. Akt is up-regulated in many human cancers, including BC. Approximately 21% of muscle-invasive BCs exhibit activating PI3K/Akt mutations, and another 30% display evidence of PTEN inactivation [11,12]. Stimulation of the PI3K/Akt/PTEN signaling pathway via aberrant RTK activity, which occurs by the reversible alteration of the phosphorylation state of specific tyrosine residues, results in the impairment of cell survival/proliferative responses [13,14]. The RTK-mediated activation of PI3K increases the phosphatidylinositol 3,4,5 phosphate (PIP_3_) levels, leading to Akt activation [15]. The lipid phosphatase PTEN acts as a negative regulator of the PI3K/Akt pathway by dephosphorylating PIP. Phosphorylated PTEN is unable to associate with the membrane due to a conformational change and, hence, does not inhibit PI3K signaling [16]. The balance between PTEN and PI3K signaling modulates the basal PIP_3_ levels in the plasma membrane, which, in turn, regulates cell survival and proliferation [16].

Lysosomes and cathepsins play a major role in tumor cell death [17,18]. The lysosome permeabilization or rupture, facilitates cathepsin release, causing cell death via mitochondria-dependent apoptosis [19,20]. Among lysosomal enzymes, cathepsin B (CB) is a cysteine protease primarily involved in the degradation or processing of lysosomal proteins [21], as well as vesicle trafficking [22], inflammasome generation [23] and cell death [24].

In the present study, we found that sorafenib induces apoptosis of BC cells via the activation and inactivation of PTEN and Akt pathways, respectively, thereby stimulating CB activity and Bid fragmentation, which induced the mitochondrial-dependent pathway of apoptosis. In addition, in the view to reduce the sorafenib-mediated side effects and to maintain the efficiency of drug treatment, our findings provided a rationale for the use of the Akt inhibitor perifosine as an adjuvant with sorafenib in clinical trials of advanced BC.

## RESULTS

### Sorafenib inhibits the survival and induces apoptosis in BC cells

**Figure 1:**
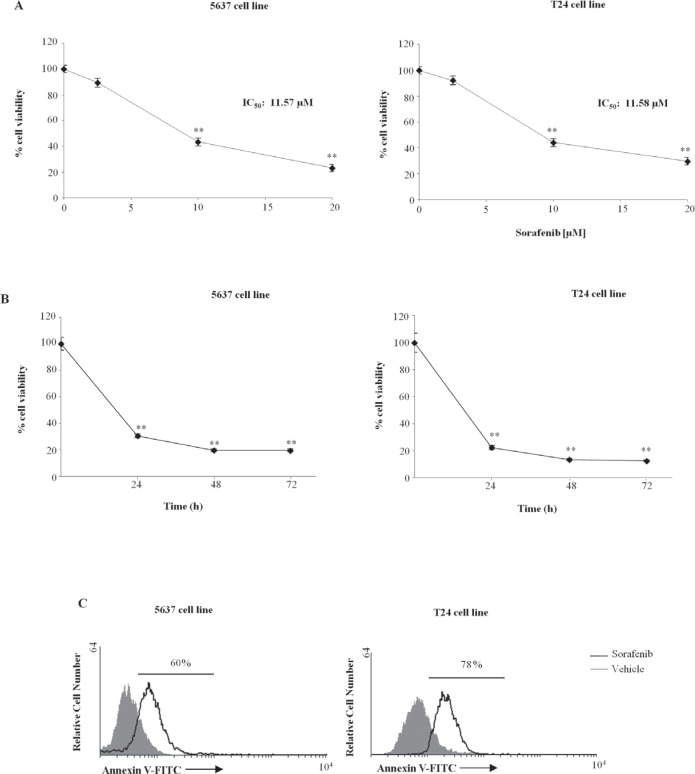
Sorafenib reduces the 5637 and T24 cell viability and induces apoptotic cell death A) Cell growth was evaluated by MTT assay in 5637 and T24 cells treated with different doses of sorafenib (2.5, 10, 20 μM) for 24 h. Data shown are the mean ± SD of three independent experiments. **p<0.01, sorafenib- vs vehicle-treated cells. IC value was determined as concentration exerting a half-maximal inhibition of cell growth. B) Cell growth was evaluated by MTT assay in 5637 and BC T24 cells treated with sorafenib (20 μM) for different times (24, 48 and 72 h). Data shown are the mean ± SD of three independent experiments. **p<0.01, vs vehicle-treated cells. C) 5637 and T24 cells treated with sorafenib 20 μM or with vehicle for 24 h were stained with Ann V-FITC and analyzed by FACS. One representative experiment out of three independent experiments is shown.

The ability of sorafenib to inhibit the viability of the 5637 and T24 BC cell lines was evaluated through dose-response (2.5, 10 and 20 μM) (Fig. 1A) and time-course (24, 48 and 72 h) (Fig. 1B) analysis. Sorafenib inhibited the growth of BC cells at 24 h after treatment, with IC_50_ values of 11.57 μM and 11.58 μM for 5637 and T24 BC cells, respectively.

Then we investigated by FITC-conjugated Annexin V (Ann V-FITC/PI) staining and flow cytometric analysis, the ability of sorafenib to induce apoptosis of BC. We found that sorafenib at 10 μM and 20 μM (Supplementary Figure 1) induces dose-dependent apoptosis of both 5637 and T24 BC cells, with 20 μM showing higher apoptotic effects compared with dose 10 μM; the 20 μM sorafenib-induced effect started at 12 h (% apoptotic cells: 52% and 62% in 5637 and T24 BC cells, respectively) and was maximal at 24 h after treatment (% apoptotic cells: 60% and 78% in 5637 and T24 BC cells, respectively) (Fig. 1C and supplementary Fig S1).

### Sorafenib induces lysosome permeabilization, cathepsin B (CB) release and activation in BC cells

**Figure 2:**
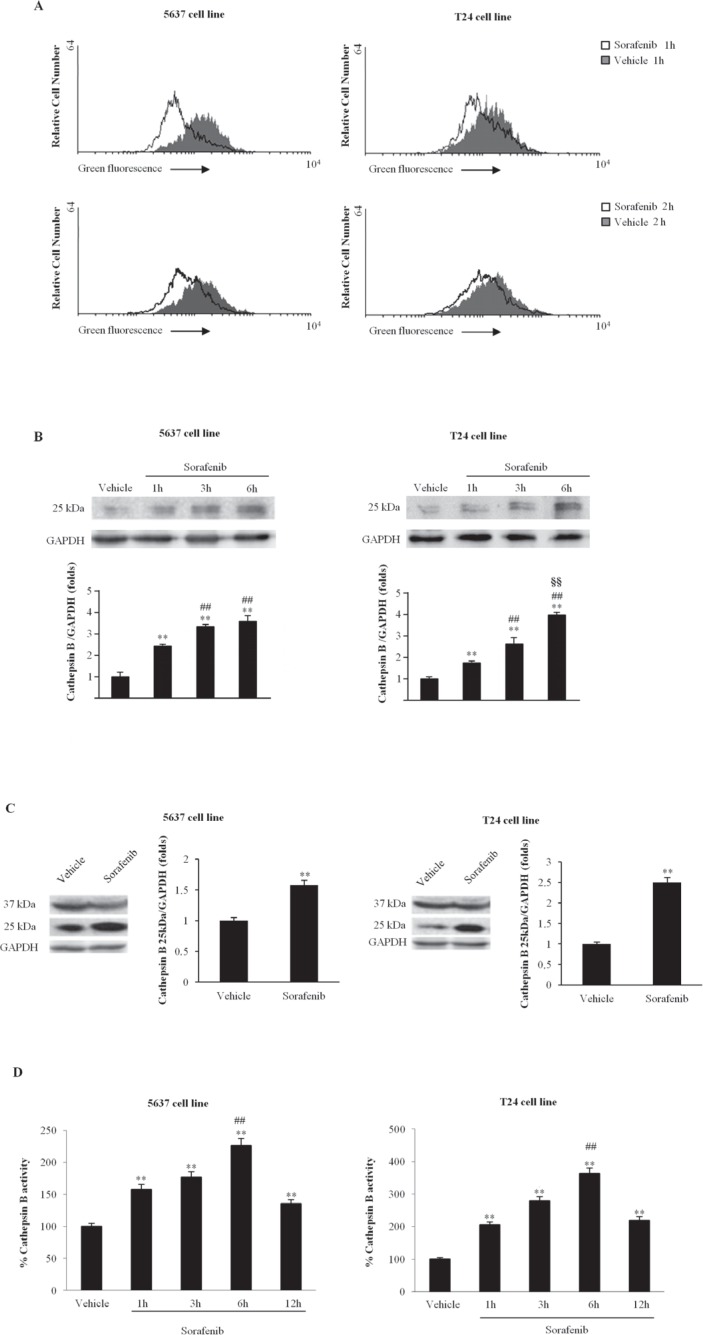
Sorafenib induces lysosome permeabilization and CB activation A) Lysosome leakage was evaluated in BC cells treated with sorafenib (20 μM) or vehicle for 1 and 2h by lysotracker staining. One representative experiment out of three independent experiments is shown. B) Representative blots of cytosol extracts from BC cells treated with sorafenib (20 μM) for different times or with vehicle for 1h probed using an anti-CB Ab. The GAPDH expression levels were used as a loading control. The densitometric data are expressed as the mean ± SD of three independent experiments. **p<0.01 vs vehicle-treated cells; ^##^p<0.01 vs 1 h of treatment; § §p<0.01 vs 3 h of treatment. No statistical significant difference was found between untreated and vehicle-treated cells or comparing different times of vehicle-treatment each other (data not shown). C) Lysates from 5637 and T24 cells treated for 1h with sorafenib (20 μM) or vehicle were separated via 12% SDS-PAGE and probed using an anti-CB Ab. The GAPDH expression levels were used as a loading control. One representative experiment is shown. The densitometric data are expressed as the mean ± SD of three independent experiments. **p<0.01 vs vehicle-treated cells. No statistical significant difference was found between untreated and vehicle-treated cells (data not shown). D) CB activity was measured in 5637 and T24 cells treated for different periods with sorafenib (20 μM) or for 1 h with vehicle using the fluorogenic peptide Z-Arg-Arg-AMC and a SpectraMax Gemini XPS microplate reader. The presented data are expressed as the means ± SD of three independent experiments. **p<0.01 vs vehicle-treated cells; ^##^p<0.01 vs 1, 3 or 12 h of treatment. No statistical significant difference was found between untreated and vehicle-treated cells or comparing different times of vehicle-treatment each other (data not shown).

Several studies have suggested a close association between lysosomal function and apoptosis [25]. Moreover, CB has been demonstrated to be one of the major lysosomal cysteine proteases that plays an important role in apoptosis [26]. Thus, the integrity of lysosome in BC cells, treated with sorafenib, was evaluated by Lysotracker Green staining and cytofluorimetric analysis. Lysotracker was added to BC cells treated for 1 and 2 h with sorafenib, and green fluorescence was investigated using the FL1 channel. We found that, due to loss of lysosome integrity, sorafenib mainly reduces the green fluorescence at 1 h after treatment of about 50% and 30% in T24 and 5637 BC cells, respectively (Fig. 2A). Then, to further address the release of lysosomal CB into the cytosol of BC cells, a cytosolic extract from BC cells treated for different times (1, 3 and 6 h) with sorafenib, obtained eliminating cytosolic organelles by ultracentrifugation, was analyzed by immunoblotting. We observed that in sorafenib-treated BC cells, the CB level increases in a time-dependent manner; it starts at 1 h and is maximal at 6 h after treatment (Fig 2B).

In addition, we investigated, by using whole cell lysates, the ability of sorafenib to trigger CB activation and enzymatic activity in BC cells. We found that sorafenib treatment increases CB activation at 1 h after treatment by stimulating the cleavage of the immature CB pro-peptide (p37) to generate its active form (p25) (Fig. 2C). Moreover, by the use of the fluorogenic peptide Z-Arg-Arg-AMC we also found that sorafenib enhances CB activity in a time-dependent manner; CB activity was evidenced at 1 h and peaks at 6 h after treatment (Fig. 2D). Overall, these results evidenced that sorafenib induces lysosome leakage, CB release, activation and enzymatic activity in BC cells.

### CB directly interacts with sorafenib

**Figure 3:**
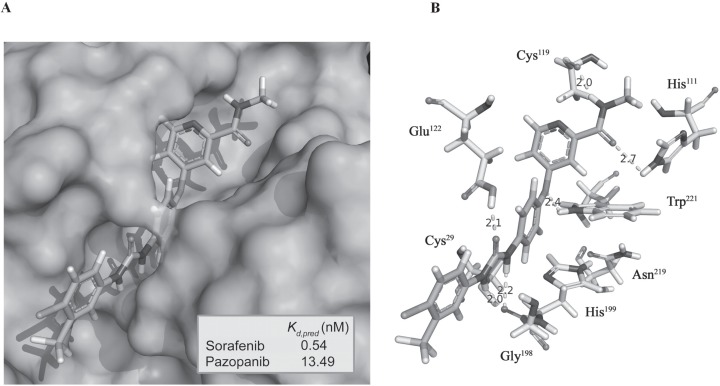
The catalytic groove of CB interacts with sorafenib A) Sorafenib docks in the catalytic groove of CB (represented with protein surface) and covers the active site. The predicted equilibrium dissociation constant (K,_d pred_) of the sorafenib/CB complex compared with that of pazopanib/CB is shown in the box. B) Cys29 and the other amino acids of CB involved in H-bond formation with the drug are indicated.

We demonstrated the feasibility of a molecular interaction between CB and sorafenib and by performing molecular modeling of the docking site of the potential CB/sorafenib complex. The analysis of molecular docking between CB and sorafenib revealed complete insertion of this drug into the catalytic groove of CB, covering the active site (Cys29, His199 and Asn219). In particular, sorafenib bound to CB with a strong affinity (0.54 nM), forming a complex displaying a total intermolecular energy of −95.35 kcal/mol (E_vaw_ = −77.87 kcal/mol; E_Elect_) = −17.48 kcal/mol) (Fig. 3 A, B). Notably, this predicted affinity is equivalent to that of the physiological inhibitor cystatin C (0.6nM) [27]. Specifically, the CB/sorafenib complex contained 6 H-bonds established via the amino acids His111, Cys119, Glu122, Gly198 and Trp221 (mean length of 2.23Å) that contribute to complex stabilization, along with electrostatic and van der Waals forces. As for the interaction between CB and other TKIs [28], CB binds to pazopanib with an affinity of 13.49 nM, thus forming a complex displaying a total intermolecular energy of −85.20 kcal/mol (E_vaw_= −79.14 kcal/mol; E_Elect_ = −6.06 kcal/mol) (Supplementary Fig. 2 A, B). Collectively, the results of molecular modeling and the comparative analysis with pazopanib strongly suggest that there is a direct interaction between CB and sorafenib that forms a stable complex.

### Sorafenib, through CB activation, induces Bid activation that triggers the intrinsic pathway of apoptosis in BC cells

**Figure 4:**
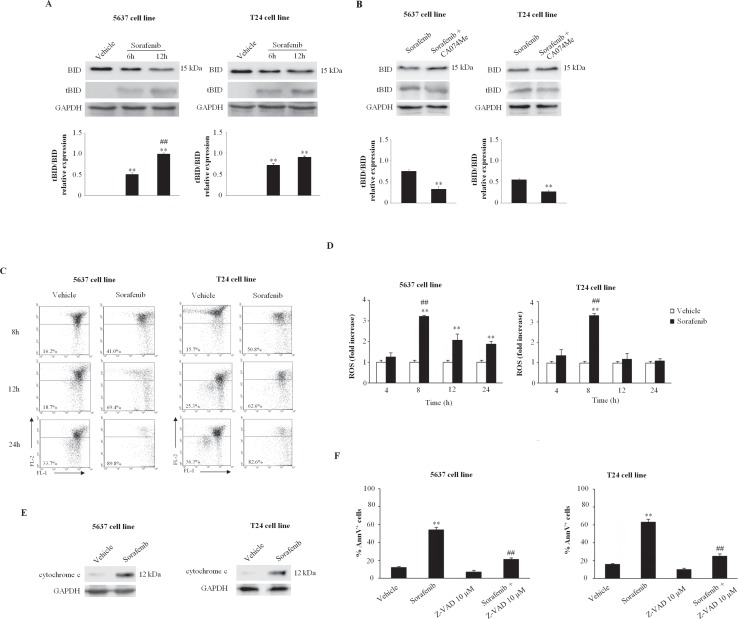
Sorafenib induces BID cleavage and mitochondrial-dependent apoptosis in 5637 and T24 BC cells A) Lysates from 5637 and T24 cells treated for 6 or 12 h with sorafenib (20 μM) or for 6 h with vehicle were separated via 12% SDS-PAGE and probed using an anti-Bid or anti-GAPDH Abs. One representative experiment out of three independent experiments is shown. The densitometric data are expressed as the mean ± SD of three independent experiments. **p<0.01 vs vehicle-treated cells; ^##^p<0.01 vs 6 h of treatment. No statistical significant difference was found between untreated and vehicle-treated cells or comparing different times of vehicle-treatment each other (data not shown). B) Lysates from 5637 and T24 cells treated for 6 h with sorafenib (20 μM) alone or in combination with CA074Me were separated via 12% SDS-PAGE and probed using an anti-Bid or anti-GAPDH Abs. One representative experiment is shown. The densitometric data are expressed as the mean ± SD of three independent experiments. **p<0.01 vs sorafenib-treated cells. C) ßΨm changes on 5637 and T24 BC cells, treated with sorafenib (20 μM) or vehicle, was evaluated at different times by JC-1 staining and biparametric FL1(green)/FL2(red) flow cytometric analysis. Data represent one out of three separate experiments. Numbers are the percentage of cells showing a drop of ßΨm related fluorescence intensity. D) ROS production was evaluated in 5637 and T24 BC cells treated for different periods with sorafenib (20 μM) or vehicle via DCFDA staining and FACS analysis. The data are presented as the mean ± SD of three independent experiments and are expressed as the fold-change relative to the vehicle-treated cell. **p<0.01 vs vehicle-treated cells; ^##^p<0.01 vs 4, 12 or 24 h of treatment. E) Cytosolic extracts from 5637 and T24 BC cells treated for 6 h with sorafenib (20 μM) or vehicle were separated via 12% SDS-PAGE and probed using an anti-cytochrome c Ab. The GAPDH expression levels were used as a loading control. One representative experiment out of three independent experiments is shown. F) 5637 and T24 BC cells treated for 12 h with sorafenib (20 μM) alone or in combination with (10 μM) Z-VAD-FMK were stained with Ann V-FITC and analyzed via FACS. The data are expressed as the percentages of positive cells ± SD based on three separate experiments. **p<0.01 vs vehicle-treated cells; ^##^p<0.01 vs sorafenib-treated cells. Data shown are relative to T24 cell line and are representative of BC lines analyzed.

**Figure 5:**
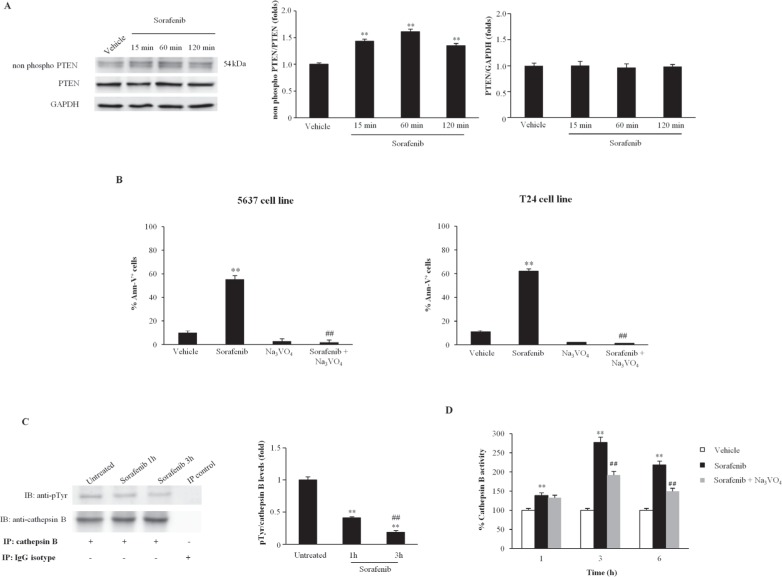
Sorafenib stimulates CB dephosphorylation and PTEN activity in T24 BC cells A) Lysates from T24 cells treated with sorafenib (20 μM) for 15, 60 and 120 min or with vehicle for 15 min were separated via 9% SDS-PAGE and probed using anti-non-phospho-PTEN, anti-PTEN and anti-GAPDH Abs. One representative experiment is shown. The densitometric data are expressed as the mean ± SD of three separate experiments. **p<0.01 vs vehicle-treated cells. No statistical significant difference was found between untreated and vehicle-treated cells or comparing different times of vehicle-treatment each other (data not shown). B) T24 BC cells treated for 12 h with sorafenib (20 μM), alone or in combination with orthovanadate (Na_3_ VO_4_ 0.25 mM) were stained with Ann V-FITC and analyzed via FACS. The data are expressed as the percentage of positive cells and are presented as the means ± SD of three independent experiments. **p<0.01 vs vehicle-treated cells; ^##^p<0.01 vs sorafenib-treated cells. C) Cell lysates from T24 BC cells either untreated or treated with sorafenib (20 μM) for 1 or 3 h were immunoprecipitated (IP) using a monoclonal anti-CB Ab. The immunoprecipitates were separated via 12% SDS-PAGE and then immunoblotted (IB) using Abs against phospho-tyrosine (anti-pTyr) and CB. The lysates were also immunoprecipitated using isotype-matched IgG, which was used as a control. One representative experiment is shown. The densitometric data are expressed as the mean ± SD of three separate experiments. **p <0.01 vs untreated cells; ^##^p<0.01 vs 1 h of treatment. D) CB activity was measured in T24 BC cells treated for different periods with vehicle or sorafenib (20 μM) either alone or in combination with orthovanadate (Na_3_ VO_4_, 0.25 mM). The data are presented as the means ± SD of three independent experiments. **p<0.01 vs vehicle-treated cells; ^##^p<0.01 vs sorafenib-treated cells. Data shown are relative to T24 cell line and are representative of BC lines analyzed.

CB has been found to activate the conversion of Bid to its active form tBid and consequently to trigger the intrinsic pathway of apoptosis [29]. Thus, the effect of sorafenib on Bid activation was evaluated. We found that treatment with sorafenib (20 μM) for 6 and 12 h induces the cleavage of the pro-apoptotic Bid protein to generate the truncated form tBid, resulting in a reduction in Bid (p22) expression and a parallel increase in tBid (p15) expression (Fig. 4A). This effect was markedly reverted at 6 h after sorafenib treatment by the CB inhibitor, CA074Me used at 2.5 μM (Fig. 4B) and 5 μM (data not shown). Next, the involvement of mitochondria and ROS generation in the sorafenib-induced apoptosis of BC cells was evaluated via JC-1 and DCFDA staining and FACS analysis. We found that sorafenib induces a time-dependent decrease in ßΨm and a parallel increase in ROS production in both types of BC cells. Mitochondrial depolarization was detected approximately at 8 h and peaking at 24 h after treatment (89.8% in 5637 cells; 82.6% in T24 BC cells) (Fig. 4C). We also observed that sorafenib stimulates ROS generation that peaks at 8 h and declines at the later time point (Fig. 4D). In addition, by western blot analysis of sorafenib-treated cytosol extracts of BC cells, we found that sorafenib induces the translocation of cytochrome c from the mitochondria to the cytosol of BC cells (Fig. 4E). Finally, we further examined the caspase-dependence of sorafenib-induced apoptosis. Thus, the BC cells were treated for 12 h with 10 μM (Fig. 4E) and 50 μM (data not shown) of the pan-caspase inhibitor, the ZVAD-fmk peptide alone or in combination with sorafenib. A significant reduction in the percentage of apoptotic Ann V^+^ cells was detected in BC cells treated with sorafenib (20 μM) and ZVAD-fmk (10, 50 μM) compared with those cells treated with sorafenib alone (Fig. 4F).

Collectively, these results indicate that, sorafenib, activating CB, stimulates BID cleavage and promotes the activation of the mitochondrial-dependent intrinsic pathway of apoptosis in BC cells.

### Role of PTEN in sorafenib-induced CB activation in BC cells

Sorafenib has been shown to activate various PTPs in different cell types [14,30-32]. Herein, the effect of sorafenib on SHP-1 and/or PTEN activation was evaluated in BC cells via western blot analysis. We found that although SHP-1 is expressed in T24 BC cells, sorafenib does not induce SHP-1 phosphorylation at any time point after sorafenib treatment (Supplementary Fig. 3). In addition, sorafenib significantly increased PTEN phosphatase activity in a time-dependent manner, as shown by the increase in the non-phosphorylated PTEN levels in T24 cells. PTEN dephosphorylation was initially detected at 15 min, progressively increasing until 1 h and slightly decreasing at 2 h after sorafenib treatment (Fig. 5A). No changes in the total PTEN protein levels were detected in the sorafenib-treated T24 BC cells. Similar results were obtained using 5637 BC cells (data not shown). Pretreatment of BC cells with 0.25 mM (Fig. 5B) and 0.50 mM (data not shown) of the specific tyrosine-phosphatase inhibitor, orthovanadate for 30 min prior to treatment with sorafenib (20 μM) strongly reduced at 12 h after treatment, the percentage of sorafenib-induced Ann V^+^ apoptotic cells, as evaluated by FACS analysis (Fig. 5B).

Recent report showed that the increase of CB activity is related to its rapid tyrosine-dephosphorylation [33]. Thus, the ability of sorafenib to reduce CB tyrosine phosphorylation levels was evaluated. Based on immunoprecipitation and western blot analyses using anti-pTyr and anti-CB Abs, we demonstrated that in BC cells, CB is tyrosine phosphorylated at a basal level and that treatment with sorafenib decreases the CB tyrosine phosphorylation levels in a time-dependent manner (% of decrease: 60% and 75% at 1 h and 3 h, respectively) (Fig. 5C). Moreover, pretreatment of sorafenib-treated BC cells with orthovanadate markedly reduced CB activity in a time-dependent manner (Fig. 5D). Similar results were obtained using 5637 BC cells (data not shown). Taken together, these data indicate that sorafenib increases CB activity by inducing CB tyrosine dephosphorylation and this effect is likely mediated by PTEN activation.

### Sorafenib suppresses Akt tyrosine kinase activity by promoting Akt degradation in BC cells

**Figure 6:**
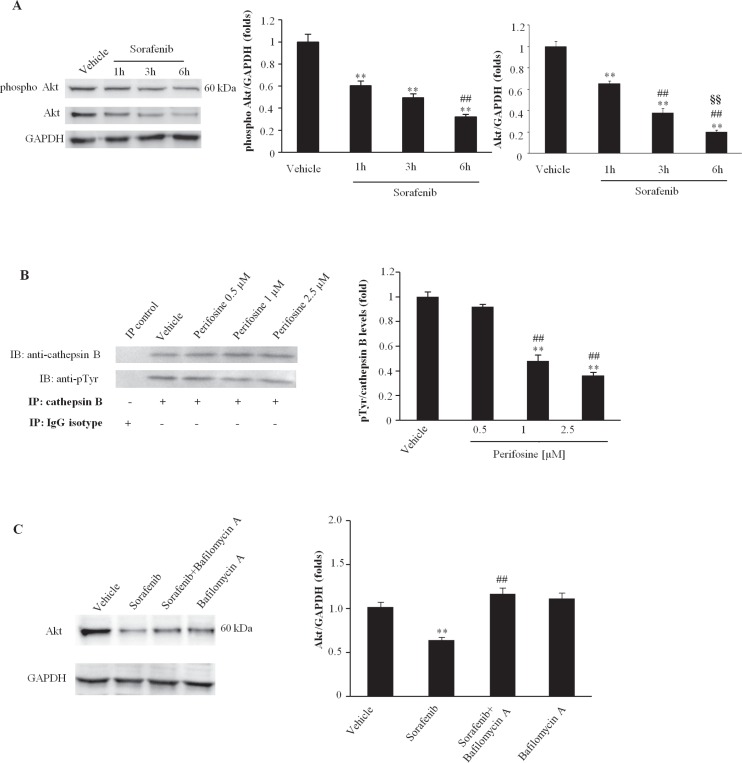
Sorafenib induces degradation of the Akt protein in T24 BC cells A) In the representative immunoblots lysates from T24 cells treated with sorafenib (20 μM) for different times or with vehicle for 1h were probed using anti-phospho-Akt, anti-Akt and anti-GAPDH Abs. The densitometric data are expressed as the mean ± SD of three separate experiments. **p<0.01 vs vehicle-treated cells; ^##^p<0.01 vs 1 h of treatment; ^§ §^p<0.01 vs 3 h of treatment. No statistical significant difference was found between untreated and vehicle-treated cells or comparing different times of vehicle-treatment each other (data not shown). B) Cell lysates from T24 cells treated for 3h with vehicle or perifosine (0.5, 1 or 2.5 μM) were immunoprecipitated (IP) using a monoclonal anti-CB Ab. The immunoprecipitates were separated via 12% SDS-PAGE and then immunoblotted (IB) using Abs against phospho-tyrosine (pTyr) and CB. The lysates were also immunoprecipitated using isotype-matched IgG, which was used as a control. One representative experiment is shown. The densitometric data are expressed as the mean ± SD of three separate experiments. **p <0.01 vs vehicle-treated cells; ^##^p<0.01 vs 0.5 μM of perifosine. No statistical significant difference was found between untreated and vehicle-treated cells (data not shown). C) Lysates from T24 cells treated for 6 h with vehicle or sorafenib (20 μM) and bafilomycin A1 (50 nM) alone or in combination were separated via 9% SDS-PAGE and probed using anti-Akt or anti-GAPDH Abs. One representative experiment is shown. The densitometric data are expressed as the mean ± SD of three separate experiments. **p<0.01 vs vehicle-treated cells; ^##^p<0.01 vs sorafenib-treated cells. No statistical significant difference was found between untreated and vehicle-treated cells or comparing different vehicles-treatment each other (data not shown). Data shown are relative to T24 cell line and are representative of BC lines analyzed.

PTEN is a negative regulator of Akt activity [16] and sorafenib has been found to induce PTEN activation. Thus, the ability of sorafenib to reduce the Akt tyrosine phosphorylation levels in T24 BC cells was evaluated. Based on western blot analysis, we demonstrated that sorafenib inhibits Akt phosphorylation in T24 BC cells in a time-dependent manner (Fig. 6A). The inhibition of phosphor-Akt expression paralleled that of the total Akt protein, indicating that in T24 BC cells, sorafenib acts inducing a time-dependent down-regulation of the Akt protein (Fig. 6A). Then, we further examined the ability of perifosine, a specific inhibitor of Akt phosphorylation, to inhibit CB tyrosine phosphorylation in T24 BC cells. We showed that at 3 h after treatment, perifosine markedly reduces the basal CB tyrosine phosphorylation levels in a dose-dependent manner (Fig. 6B). Similar results were obtained using 5637 BC cells (data not shown). Finally, to elucidate the mechanism of Akt down-regulation, BC cells were treated for 6 h with sorafenib in the presence of 50 nM (Fig. 6C) and 100 nM (data not shown) of the lysosome inhibitor, bafylomycin A (Fig. 6C). We found that bafylomycin A completely blocks sorafenib-induced Akt protein degradation. Treatment with the lysosome inhibitor in the absence of sorafenib did not induce any significant effect (Fig. 6C). Similar results were obtained using the 5637 BC cells (data not shown).

### The Akt inhibitor perifosine sensitizes BC cells to sorafenib-induced apoptotic cell death

**Figure 7:**
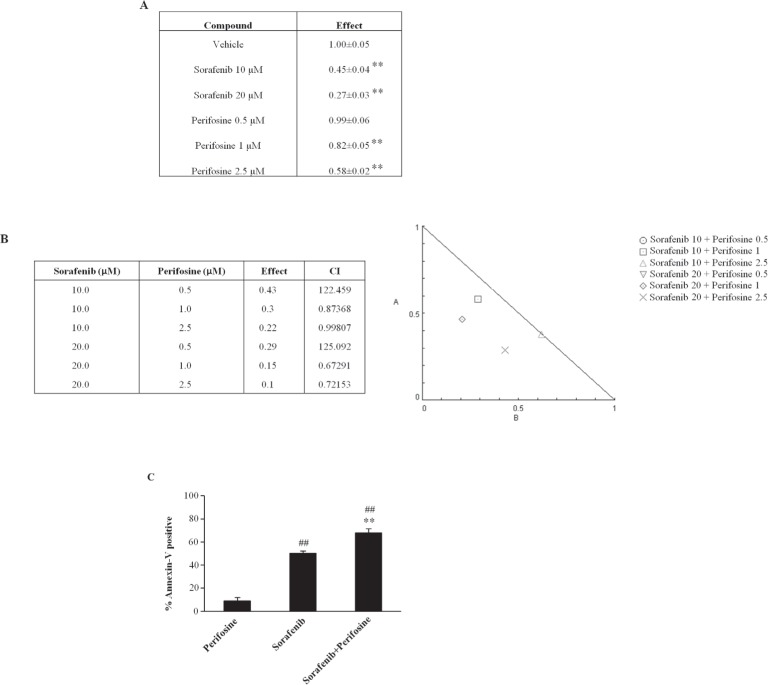
Perifosine in combination with sorafenib increases the sensitivity of T24 BC cells to the sorafenib-induced cytotoxicity A) Cell viability of T24 BC cells untreated or treated for 24h with sorafenib (10 and 20 μM) and perifosine (0.5, 1, 2.5 μM) was evaluated by MTT assay. Data shown are the mean ± SD of three independent experiments. **p<0.01 vs vehicle-treated cells; No statistical significant difference was found between untreated and vehicles-treated cells (data not shown). For sake of simplicity only one vehicle sample is shown. B) The synergistic activity of sorafenib and perifosine used in combination on the viability of T24 BC cells was determined by the isobologram and combination index (CI) methods. The CI was used to express synergism (CI<1), additivity (CI=1) or antagonism (CI>1) and was calculated according to the standard isobologram equation. C) T24 BC cells treated for 24 h with sorafenib (10 μM) and perifosine (2.5 μM) alone or in combination, were stained with Ann V-FITC and analyzed by FACS. Data, expressed as the percentage of Ann V positive cells, are the mean ± SD of three separate experiments. **p<0.01 vs sorafenib-treated cells; ^##^p<0.01 vs perifosine-treated cells. Data shown are relative to T24 cell line and are representative of BC lines analyzed.

Recent data have indicated that sorafenib in combination with perifosine induces mitochondria-mediated cell death and anti-tumor effects in NOD/SCID mice xenografted with a Hodgkin lymphoma cell line [34]. However, neither *in vitro* nor *in vivo* findings on the effects of sorafenib administered in combination with perifosine has been reported in BC cells to date. Thus, we evaluated the effects of different doses of perifosine (0.5, 1.0 or 2.5 μM) alone and in combination with sorafenib (10 and 20 μM) in T24 BC cells. We found that perifosine reduces the viability of T24 BC cells in a dose-dependent manner at 24 h, showing a maximal effect (42.1% of inhibition) with the 2.5 μM dose (Fig. 7A). By standard isobologram and CompuSyn software analysis we evaluated the combination index (CI) and we found that the combination of sorafenib 10 or 20 μM with perifosine at the doses 1 and 2.5 μM shows synergistic effect increasing the cytotoxicity against T24 BC cells (Fig. 7B). Moreover, the use of sorafenib at 10 μM in combination with perifosine at different doses (1.0 or 2.5 μM) approximates the cytotoxic effects induced by sorafenib (20 μM) alone (Fig. 7B). This synergistic effect does not depend on the direct ability of perifosine to induce apoptosis (Fig. 7C), although, the perifosine/sorafenib combination significantly increases the sorafenib-induced apoptosis of BC cells (Fig. 7C). Thus, perifosine by inducing CB activation sensitized the BC cells to sorafenib-induced apoptosis. Similar results were obtained using the 5637 BC cells (data not shown).

## DISCUSSION

Herein, we demonstrated that sorafenib treatment stimulates the intrinsic pathway of apoptosis in BC cells. Several studies have suggested a close association between lysosomal function and apoptosis [25,35-38]. Anti-cancer agents have been reported to induce lysosome membrane permeabilization (LMP) [37,39-41], or rupture [25,42] which is followed by the release of lysosomal cathepsins. It has been shown that lysosomes are particularly sensitive toward oxidative stress [43,44].

Here, we demonstrated, for the first time, that the sorafenib-induced effects are mediated by its ability to stimulate the LMP leading to release of CB into the cytosol of BC cells. Then, BID activation and release of the tBid fragment [19], mitochondrial depolarization and cytochrome c release, ROS production and caspase activation are induced, resulting in the complete execution of the intrinsic pathway of apoptosis [17,45]. Similarly, in murine (MBT2 and MB49) and human T24 BC cells, Bacillus Calmette-Guerin induces CB activation and Bid fragmentation, thereby activating the intrinsic apoptotic pathway [29]. The effect of sorafenib treatment on CB activation in BC cells was further supported by a molecular docking analysis of the molecular interaction between CB and sorafenib that indicated a complete insertion of sorafenib into the catalytic groove of CB with a strong binding affinity (25-fold higher than pazopanib). The molecular interaction between CB and sorafenib results in the formation of a stable complex. In this regard, Cathepsins have been found to form multimolecular molecular complex with different molecules such us signaling and cytoscheleton proteins and drugs, and a role for these complexes in apoptotic cell death have been provided [46]. Thus, we can hypothesize that CB also binds Akt and/or PTEN, other than sorafenib, to form a three molecular complex permitting both PTEN and CB dephosphorylation and activation. In this view, recently, it has been found that the increase in CB activity is associated with its rapid tyrosine dephosphorylation [33]. Structural analysis of CB revealed many phosphorylation sites for protein kinase C or casein kinase II, as well as a tyrosine kinase phosphorylation site [33]. In this regard, based on immunoprecipitation and western blot analysis using anti-CB and anti-pTyr Abs, respectively, we confirmed not only that CB is tyrosine phosphorylated at a basal level, but also that sorafenib treatment significantly reduces the CB phosphorylation levels. Moreover, orthovanadate, that inhibits PTEN activation, reverts the sorafenib-induced apoptosis in BC cells, suggesting that sorafenib-induced CB tyrosine dephosphorylation represents a requisite to obtain the necessary CB enzymatic activity required to stimulate apoptosis in BC cells. Previous reports have indicated that sorafenib treatment stimulates SHP-1 and/or SHP-2 tyrosine phosphatase activity in breast cancer [32], glioblastoma [30], HCC [14] and pancreatic cancer [31] and that orthovanadate reduces the sorafenib-induced inhibition of signal transducer and activator of transcription 3 (STAT3)- mediated tyrosine phosphorylation and increased PTP and SHP-2 activity in glioma cells [30]. Herein, we found that sorafenib significantly induces PTEN dephosphorylation, as evidenced by the increase in the non-phosphorylated form of PTEN relative to the total PTEN levels, and activation of its phosphatase activity [16]. Thus, the sorafenib-induced increase in CB activity results in Bid activation, thereby stimulating the apoptosis of BC cells. In addition, the CB dephosphorylation, might also occur via sorafenib-induced inhibition of Akt tyrosine kinase activity. In this regard, sorafenib reduces the Akt phosphorylation by inducing the degradation of the Akt protein. Pretreatment of BC cells with the lysosomal enzyme inhibitor bafilomycin A completely abrogated the sorafenib-induced Akt degradation, demonstrating that sorafenib acts at a post-transcriptional level by inducing lysosome-dependent degradation of the Akt protein [47]. Similarly, sorafenib has been demonstrated to promote the lysosomal degradation of RET protein in HEK293 cells [48] and bafilomycin A inhibits the sorafenib-induced degradation of ATP-binding cassette sub-family G member 2 transporters (ABCG2) in ABCG2-transfected HEK293 cells [49]. Collectively, our data demonstrated that sorafenib treatment by inducing PTEN activation and Akt inactivation/degradation, increases the CB dephosphorylation and enzymatic activity, thus stimulating BC cell apoptosis.

Perifosine is an oral Akt inhibitor, currently evaluated in phase III clinical trials, shown to inhibit Akt and mitogen-activated protein kinase (MAPK) phosphorylation in prostate PC3 cells, multiple myeloma, HCC, glioma cells and renal carcinoma [50-54]. In addition in lymphoma cells, the perifosine is used in combination with sorafenib [34]. No data on the ability of perifosine to increase sorafenib-mediated cytotoxicity in BC cells has been reported so far. By isobologram analysis we found that sorafenib (10 μM) in combination with perifosine (2.5 μM), synergize to increase the cytotoxicity against T24 BC cells. The effect of sorafenib/perifosine drug combination in T24 BC cells, does not depend on the ability of perifosine to directly stimulate the apoptosis, but also to increase the CB activation by inducing dephosphorylation and activation of CB, thus sensitizing BC cells to sorafenib-induced apoptosis.

The knowledge of the molecular mechanisms of the direct anti-tumor effect of sorafenib could permit to identify new therapeutic targets to improve the care of advanced bladder cancer patients.

Overall, in this study we demonstrated that activation of PTEN- and the inactivation of Akt-mediated-pathways by sorafenib stimulates the CB-dependent apoptosis of BC cells. Finally, the “dual target therapy” with perifosine and low dose of sorafenib in combination, by coupling the effects of sorafenib on PTEN and Akt dephosphorylation, with that induced by perifosine on CB dephosphorylation and activation, improves the sorafenib anti-tumor effects and potentially reduce the side-effects, thus providing a rationale for the use in clinical trials in patients suffering from advanced BC.

## MATERIALS AND METHODS

### BC cell lines

The p53 mutant, 5637 and T24 BC cell lines, purchased from American Type Culture Collection (ATCC, Rockville, MD, USA), were maintained in RPMI-1640 medium (Lonza Bioresearch, Basel, Switzerland) supplemented with 10% heat-inactivated fetal bovine serum, 2.5 mM N-2-hydroxyethylpiperazine-N'-2- ethanesulfonicacid (HEPES), 2mM L-glutamine, 100 IU/ml of penicillin, 100 μg/ml of streptomycin (Lonza) at 37°C, 5% CO_2_ and 95% humidity.

### Reagents

Sorafenib was kindly provided by Bayer Pharmaceuticals. The following rabbit polyclonal antibodies (Abs) were used: anti-cytochrome C (#BV-3026-3, Medical and Biological Laboratories, Nagoya City, Japan), anti-PTEN (#9559), anti-non Ser-380/Thr 382/3836 phospho-PTEN (#9569), anti-AKT (#9227), anti-phospho AKT (#9271), anti-Src-homolog protein tyrosine phosphatase (SHP-1) (#3759) and anti-phospho SHP-1 (#8849) (1:1000, Cell Signalling Technology, Danvers, USA); anti-CB (1:250, sc-13985, Santa Cruz Biotechnology, Inc., Dallas, USA). The following mouse monoclonal Abs were used: anti-Bid (1:1000, #2002, Cell Signalling Technology), anti-CB (1μg/ml, IM27L, Merck Millipore, Darmstadt, Germany), anti-phosphotyrosine (anti-pTyr, 1:1000, 05-947, Merck Millipore), IgG2a isotype control (NBP1-96778, Novus Biologicals) and horseradish peroxidase (HRP)-conjugated anti-glyceraldehyde-3-phosphate dehydrogenase (anti-GAPDH, 1:5000, G9295, Sigma Aldrich, St. Louis, USA). HRP-conjugated goat anti-rabbit (1:2000, RPN4301) and HRP-conjugated sheep anti-mouse (1:2000, NA931V) were from GE Healthcare Bio-Sciences AB (Uppsala, Sweden). Fluorescein isothiocyanate (FITC) conjugated-Annexin V (Ann V-FITC) was from Enzo Life Sciences Inc. (Farmingdale, USA). JC-1 (10 μg/mL) and lysotracker (50 nM) were from Invitrogen (San Diego, CA, USA). 2′,7′-dichlorofluorescein diacetate (DCFDA, 10 μg/ml), dimethyl sulfoxide (DMSO, used as vehicle), propidium iodide (PI, 1 μg/ml), 3-(4,5-dimethylthiazol-2- yl)-2,5-diphenyltetrazolium bromide (MTT) and sodium orthovanadate (Na_3_ VO_4_, 0.25 mM) were from Sigma Aldrich. Bafilomycin A (50 nM) was from Labogen S.r.l (Milan, Italy). Perifosine (0.5, 1 and 2.5 μM) was purchased from Selleckchem (Houston, USA), CA074Me (CB inhibitor, 2.5 μM, Sigma Aldrich) and Z-VAD-FMK (caspase inhinitor, 10 μM) from Tocris Bioscience (Bristol, UK).

### MTT assay

The colorimetric MTT assay that measures the mitochondrial conversion of the tetrazolium salt to a blue formazan salt was used to evaluate the cell viability. Briefly, 5637 and T24 BC cells (3×10^4^/ml) were seeded into 96-well plates and cultured with different doses of sorafenib (2.5, 10, 20 μM) for 24 h at 37°C, 5% CO_2_. In some experiments, cells were treated with sorafenib 20 μM for 24, 48 and 72 h. At the end of treatment, 0.8 mg/ml of MTT was added to the samples and incubated for 3 h. Then the supernatants were discarded and coloured formazan crystals, dissolved with 100 μl/well of DMSO, were read by an enzyme-linked immunosorbent assay reader (BioTek Instruments, Winooski, USA). In addition, BC cells were treated with vehicle, sorafenib (10 μM) or with perifosine (0.5, 1 and 2.5 μM) alone or in combination for 24 h. Four replicates were used for each treatment and data were represented as the average of at least three separate experiments. IC_50_ value, representing the concentration exerting an half-maximal inhibition of cell growth, was calculated using GraphPad Prism Software.

The synergistic activity of sorafenib and perifosine used in combination on the viability of 5637 and T24 BC cells was determined by the isobologram and CI methods (CompuSin Software, ComboSyn, Inc. Paramus, NJ 2007). The CI was used to express synergism (CI<1), additivity (CI=1) or antagonism (CI>1) and was calculated according to the standard isobologram equation [55].

### Lysosome staining by LysoTracker

To investigate the involvement of lysosomes, BC cells were stained with LysoTracker-green. Briefly, 5637 and T24 cells (3×10^5^/ml), seeded into 6-well plates, were treated with sorafenib (20 μM) for 1 and 2 h. Then LysoTracker-green probe (50 nM) was added for 30 min at 37°C. Samples were analyzed by a FACScan cytofluorimeter using the CellQuest software; fluorescence intensity was expressed in arbitrary units on logarithmic scale.

### Annexin V and PI staining

Apoptotic cell death on 5637 and T24 BC cells was evaluated using Ann V-FITC and PI staining followed by biparametric FACS analysis. Briefly cells, seeded as above described, were treated with sorafenib (10 and 20 μM) or vehicle for different times and then incubated with Ann V-FITC and PI according to the manufacturer's instruction. The simultaneous staining with Ann V-FITC and the non-vital dye PI allows the discrimination of intact cells (FITC^−^PI^−^), early apoptotic (FITC^+^PI^−^) and late apoptotic or necrotic cells (FITC^+^PI^+^). The percentage of positive cells determined over 10,000 events was analyzed on a FACScan cytofluorimeter using the CellQuest software. In some experiments, T24 BC cells were pretreated for 30 min with sodium orthovanadate (0.25 mM) [56] or Z-VAD-FMK (10 μM) [28] before the treatment with sorafenib (20 μM) for 24 h. In addition, the percentage of Ann V^+^ cells was evaluated in T24 BC cells treated with sorafenib (20 μM) alone or in combination with perifosine (2.5 μM) for 12 h.

### Reactive Oxygen Species (ROS) production

5637 and T24 BC cells (4×10^4^/ml) were seeded into 24-well plates and cultured for different times (4, 8 and 12 h) with sorafenib (20 μM) or vehicle. Cells were washed with PBS, pulsed with DCFDA for 10 min at 37°C, 5% CO_2_, and analyzed by FACScan cytofluorimeter using the CellQuest software. The percentage of positive cells determined over 10,000 events was analyzed on a FACScan cytofluorimeter using the CellQuest software.

### Mitochondrial transmembrane potential (ßΨm)

ßΨm was evaluated by JC-1 staining. 5637 and T24 BC cells (4×10^4^/ml), seeded into 24-well plates, were treated with sorafenib (20 μM) or vehicle for different times and then incubated for 10 min at room temperature with JC-1. JC-1 was excited by an argon laser (488 nm); green (530 nrn)/red (>570 nrn) emission fluorescence was collected simultaneously. Carbonyl cyanide chlorophenylhydrazone protonophore, a mitochondrial uncoupler that collapses ßΨm was used as positive control (data not shown). Samples were analyzed by a FACScan cytofluorimeter using the CellQuest software; fluorescence intensity was expressed in arbitrary units on logarithmic scale

### Molecular docking analysis

Molecular docking analysis was performed on Linux Red Hat Pentium4 – based platform using Autodock Vina [57] and InsightII software (Release 2005, Accelrys Ltd., Cambridge, U.K). Human CB X-ray crystal structure (pdbID:1CSB) [58] was obtained from the Protein Data Bank [59]. Pdbqt three-dimensional structure files of sorafenib and pazopanib comparative drugs and CB were obtained by adding polar hydrogens and removing water from protein, and were submitted to Vina, setting a docking grid with a size of 60Å × 65Å × 50Å, centered at the position x = 21.18, y = −6.05, z = 38.08. Then, the most stable composite CB/TKI drug model was refined using Discover module of InsightII, minimizing all atoms as previously reported [59]. Docking module was used to calculate total intermolecular energy (E = E + E) and Ludi module was used to calculate LUDI Score and the related predicted equilibrium dissociation constants *K_d_* by the standard equation LUDI Score = −100 × log_10_ *K_d_* and the Energy Estimate 3 [58]. PyMOL software (DeLano Scientific LLC, San Carlos, CA, 2009-10) was used to render all the output from InsightII and to calculate the distances of hydrogen bonds as measured between the hydrogen and its assumed binding partner.

### Cathepsin B activity

CB proteolytic activity was measured in 5637 and T24 BC cells at 1, 3, 6 and 12 h after sorafenib (20 μM) treatment following the protocol described by Tchoupe and coworkers using the fluorogenic peptide Z-Arg-Arg-AMC at a final concentration of 50 μM. The mixture, containing 5 μg of protein lysate, was incubated in 50 mM phosphate buffer pH 6.0, 1 mM EDTA and 2 mM dithiothreitol for 1 h at 30 °C. The fluorescence of the hydrolyzed 7-amino-4-methyl-coumarin (AMC, λexc=365 nm, λem=449 nm) was detected on a SpectraMax Gemini XPS microplate reader (Molecular Devices). In some experiments, CB activity was evaluated in T24 cells pretreated for 30 min with 0.25 mM of sodium orthovanadate and then treated for 1, 3 and 6 h with sorafenib (20 μM) [60].

### Western blot

5637 and T24 BC cells, untreated or treated for different times with sorafenib (20 μM), vehicle or pretreated for 30 min with sodium orthovanadate (0.25 mM), before the treatment with sorafenib (20 μM) for 24 h, were lysed in lysis buffer (1M Tris pH 7.4, 1 M NaCl, 10 mM EGTA, 100 mM NaF, 100 mM Na V0, 100 mM phenylmethylsulfonyl fluoride, 2% deoxycholate, 100 mM EDTA, 10% Triton X-l00, 10% glycerol, 10% SDS, 0.1 M Na_4_ P_2_ 0_7_) containing protease inhibitor cocktail (Sigma-Aldrich) by using a Mixer Mill MM300 (Qiagen, Hilden, Germany). Lysates were separated on SDS polyacrylamide gel and transferred onto Hybond-C extra membranes (GE Healthcare). Blots were incubated with blocking solution and the primary Ab anti-CB followed by the appropriate HRP-conjugated secondary Abs according to the specific datasheet. In some experiments lysates coming from T24 BC cells treated for different times with (20 μM) sorafenib alone or in combination with bafilomycin A [27] or CA074Me were incubated with: anti-SHP-1, anti-pSHP-1, anti-PTEN, anti-non pPTEN, anti-AKT, anti-pAKT and anti-BID. In addition, we also evaluated the cytochrome c and CB in the cytosolic fraction of 5637 and T24 cells. Briefly, untreated or sorafenib-treated cells for different times (1, 3 and 6 h) were washed in ice-cold PBS and the resulting pellet was resuspended in 0.12 ml of lysis buffer (20 mM HEPES), 10 mM KCl, 1.5 mM MgCl, 1mM EDTA, 1 mM EGTA, 1 mM DTT and 0.1 mM PMSF) supplemented with protease inhibitor cocktail. After sitting on ice for 15 min, cells were disrupted by 60 times douncing in a mini-potter. The nuclei were pelletted at 1000g for 5 min at 4°C and the supernatants were separated and centrifuged for 40 min at 80000g. Then, supernatants loaded onto a 12% SDS–PAGE were transferred and incubated with anti-cytochrome c (0.5 μg/ml) or -CB (250 ng/mL) mAbs followed by HRP-conjugated anti-mouse (1:2000) Ab. GAPDH expression levels were used as loading control. The detection was performed using the LiteAblot ®PLUS or the LiteAblot ®TURBO (EuroClone, Milano, Italy) kits and densitometric analysis was carried out by evaluating three independent experiments by a Chemidoc using the Quantity One software (BioRad, Hercules, USA).

### Immunoprecipitation (IP)

Cell lysates (300 μg) from T24 and 5637 BC cells, untreated or treated for 1 and 3 h with sorafenib (20 μM) or for 3 h with perifosine (0.5, 1 and 2.5 μM), were immunoprecipitated using anti-CB mAb following the Immunoprecipitation Kit Dynabeads® Protein A instructions (Life Technologies, Monza, Italy). Immunoprecipitated samples were then applied on SDS-PAGE, transferred, and blotted with anti-pTyr and with polyclonal anti-CB Abs according to the datasheet. As IP control, isotype-matched IgG was used. Detection was performed as described above.

### Statistical analysis

The statistical significance was determined by Student's t-test and by one way ANOVA. No statistical significant difference was found between untreated and vehicle (DMSO)-treated cells or comparing different times of vehicle-treatment each other (data not shown).

## SUPPLEMENTARY FIGURES


