# Supplementary material for: Sorafenib induces cathepsin B-mediated apoptosis of bladder cancer cells by regulating the Akt/PTEN pathway. The Akt inhibitor, perifosine, enhances the sorafenib-induced cytotoxicity against bladder cancer cells
Source: Oncoscience. 2015 Mar 23;2(4):395–409. doi: 10.18632/oncoscience.147 (PMC4468325; doi:10.18632/oncoscience.147)
Supplement: Supplementary file 1 [file oncoscience-02-0395-s001.pdf]

Sorafenib induces cathepsin B-mediated apoptosis of bladder cancer cells by regulating the Akt/PTEN pathway. The Akt inhibitor, perifosine, enhances the sorafenib-induced cytotoxicity against bladder cancer cells

A

**5637 cell line**	Vehicle	Sorafenib 12h	Sorafenib 24h
PI^+	0.07 ± 0.04	1.07 ± 0.0	1.21± 0.9
$AnnV^+$ PI^+	0.52 ± 0.07	0.09± 1.3	8.86 ± 2.2 **##
$AnnV^-$ PI^-	98.46 ± 2.1	64.63 ± 1.6 **	44.14 ± 1.6 **##
$AnnV^+$	0.95± 0.5	34.21 ± 2.4 **	45.79 ± 1.7**##

**T24 cell line**	Vehicle	Sorafenib 12h	Sorafenib 24h
PI^+	0.09 ± 0.0	0.73 ± 0.0	2.06 ± 0.7
$AnnV^+$ PI^+	1.02 ± 0.5	0.76 ± 0.9	11.17 ± 2.7**##
$AnnV^-$ PI^-	97.55 ± 2.4	59.62 ± 1.2 **	40.54 ± 1.3**##
$AnnV^+$	1.34 ± 0.3	38.89 ± 1.8 **	46.23 ± 2.4**##

B

**5637 cell line**	Vehicle	Sorafenib 12h	Sorafenib 24h
PI^+	0.07 ± 0.0	0.00 ± 0.0	2.31 ± 0.3
$AnnV^+$ PI^+	0.63 ± 0.0	0.39 ± 0.0	15.63 ± 1.6 **##
$AnnV^-$ PI^-	93.84 ± 1.9	47.73 ± 1.8 **	36.74 ± 1.9 **##
$AnnV^+$	5.46 ± 0.3	51.88 ± 2.1 **	45.32 ± 1.4**##

**T24 cell line**	Vehicle	Sorafenib 12h	Sorafenib 24h
PI^+	0.01 ± 0.0	0.00 ± 0.0	3.01 ± 0.4
$AnnV^+$ PI^+	0.02 ± 0.0	0.23 ± 0.0	25.14 ± 1.1 **##
$AnnV^-$ PI^-	96.79 ± 2.2	37.34 ± 0.9 **	18.34 ± 0.7 **##
$AnnV^+$	3.18 ± 0.2	62.43 ± 1.7 **	53.51 ± 1.5 **##

Supplementary Figure 1: A,B) 5637 and T24 cells treated with sorafenib (10 µM panel A and 20 µM panel B) or with vehicle for 12, 24 h, were stained with Ann V-FITC and PI and

analyzed by FACS. Data are expressed as percentage of positive cells ± SD of three separate experiments. $^{**}p<0.01$ vs vehicle-treated cells; $^{\#\#}p<0.01$ vs 12h of sorafenib treatment. No statistical significant difference was found between untreated and vehicle-treated cells or comparing different times of vehicle-treatment each other (data not shown).

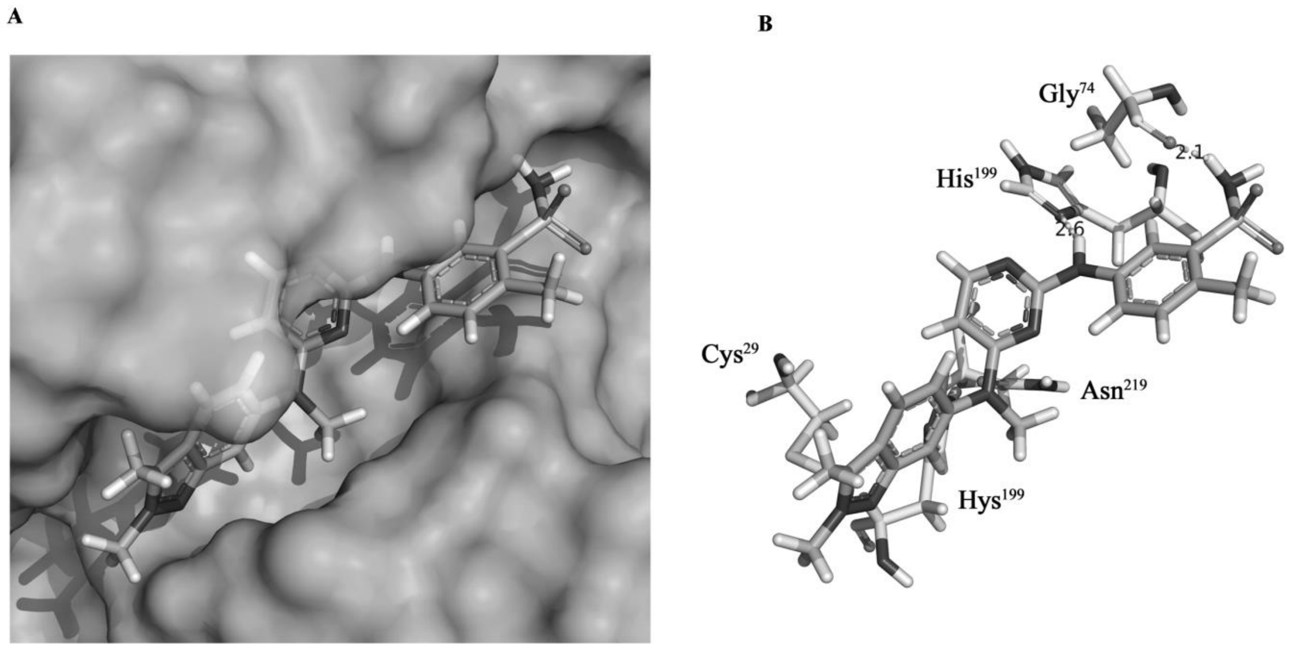

Supplementary Figure 2: Pazopanib docked into the catalytic groove of CB.

A) Pazopanib/CB complex is showed indicating the active site (light pink of the protein surface) inside the catalytic groove.

B) Cys29 and the amino acids involved in the H-bond formation.

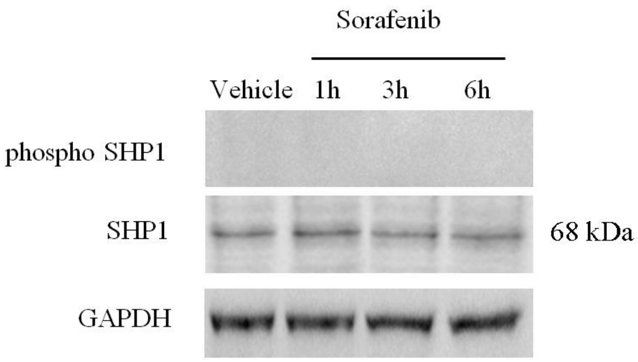

Supplementary Figure 3: Sorafenib does not modulate phosphorylation levels of SHP-1 in T24 BC cells. Lysates from T24 BC cells treated with sorafenib (20 μM) for 1, 3 or 6 h or with vehicle for 1 h were separated via 9% SDS-PAGE and probed using anti-phospho-SHP-1, anti-SHP-1 and anti-GAPDH Abs. One representative experiment out of three independent experiments is shown. No statistical significant difference was found between untreated and vehicle-treated cells or comparing different vehicles-treatment each other (data not shown).
Data shown are relative to T24 cell line and are representative of BC lines analyzed.
